# The response to receiving phenotypic and genetic coronary heart disease risk scores and lifestyle advice – a qualitative study

**DOI:** 10.1186/s12889-016-3867-2

**Published:** 2016-12-03

**Authors:** Guy Shefer, Barbora Silarova, Juliet Usher-Smith, Simon Griffin

**Affiliations:** 1 MRC- Epidemiology, University of Cambridge, 7 Cavesson Court, Cambridge, CB43TB UK; 2 Department of Public Helath and Primary Care, University of Cambridge, Cambridge, UK

**Keywords:** Health behaviour, Cardiovascular disease, Genetics, Physical activity, Risk assessment

## Abstract

**Background:**

Individuals routinely receive information about their risk of coronary heart disease (CHD) based on traditional risk factors as part of their primary care. We are also able to calculate individual’s risk of CHD based on their genetic information and at present genetic testing for common diseases is available to the public. Due to the limitations in previous studies further understanding is needed about the impact of the risk information on individual’s well-being and health-behaviour. We aimed to explore the short term response to receiving different forms of CHD risk information and lifestyle advice for risk reduction.

**Methods:**

We conducted fourty-one face-to-face interviews and two focus groups across England with participants from the INFORM trial who received a combination of individualised phenotypic and genotypic CHD risk scores and web-based lifestyle advice. Risk scores were presented in different formats, e.g. absolute 10 year risk was presented as a thermometer and expressed as a percentage, natural frequency and ‘heart age’. Interviews and focus groups explored participants’ understanding and reaction to the risk scores and attempts to change lifestyle during the intervention. We tape-recorded and transcribed the interviews and focus groups and analysed them using thematic analysis.

**Results:**

Three main themes were identified: limitations of risk scores to generate concern about CHD risk; the advantages of the ‘heart age’ format of risk score presentation in communicating a message of sub-optimal lifestyle; and intentions and attempts to make moderate lifestyle changes which were prompted by the web-based lifestyle advice.

**Conclusions:**

There are a number of limitations to the use of risk scores to communicate a message about the need for a lifestyle change. Of the formats used, the ‘heart age’, if noticed, appears to convey the most powerful message about how far from optimal risk an individual person is. An interactive, user friendly, goal setting based lifestyle website can act as a trigger to initiate moderate lifestyle changes, regardless of concerns about risk scores.

**Trial registration:**

Current Controlled Trials ISRCTN17721237. Registered 12 January 2015.

**Electronic supplementary material:**

The online version of this article (doi:10.1186/s12889-016-3867-2) contains supplementary material, which is available to authorized users.

## Background

Cardiovascular disease (CVD) is the leading cause of death globally [1], responsible for a third of all deaths [2]. Approximately 17.3 million people worldwide died from CVD in 2013 [3]. It is estimated that by 2030, 23.3 million people per year will die as a result of CVD [4]. Many risk factors, such as smoking, an unhealthy diet, and lack of physical activity, are modifiable and therefore effective preventive strategies targeting these factors can lead to a reduction in the frequency of CVD [5, 6]. Even modest changes in lifestyle, if adopted by a significant proportion of the population, have the potential to reduce global CVD by half [7].

One widely adopted preventive strategy is the assessment of CVD risk of individuals. CVD risk information is used by physicians to inform decisions about treatment, and is given to patients to encourage lifestyle changes, including taking appropriate medication in order to reduce CVD risk [8–12]. There are many different ways to communicate risk information. Numerical expressions may include percentages, natural frequencies and numbers needed to treat while graphical presentations include bar graphs and pictograms or icon arrays [13]. In an attempt to develop a more intuitive and easy to understand method of presenting CVD risk, the format of ‘heart age’ [14], sometime known as ‘vascular age’ [15] was developed. Although different studies have defined and measured ‘heart age’ differently [15], most commonly an individual’s ‘heart age’ is the age in which someone with healthy risk factors will have (according to existing population reference values) the same CVD risk which this individual has now.

Systematic reviews of randomised controlled trials and before-and-after studies have shown that the provision of phenotypic CVD risk information improves people’s accuracy of perceived risk [16, 17] but has little effect on lifestyle [16]. There are very few studies exploring the mechanism for this lack of effect on lifestyle. Theories of health behaviour change such as Health Belief model [18], Protection Motivation Theory [19], or the Theory of Planned Behaviour [20] refer to elements of perceived risk or threat as one link in a chain of thoughts and reactions that may lead to behaviour change. However, while in some of the early theories such as the Health Belief model the perception of the risk was considered a key element likely to lead to process of change, most theories now suggest that many other determinates can affect, induce or prevent different stages of the creation of intentions to change and then actual change [21, 22].

There are several additional explanations for this lack of effect of provision of risk information on lifestyle change. Firstly, many of the previous studies were small and relied on imprecise self-report measures [13]; secondly due to limitations in previous studies the optimal format for communication of CVD risk is not known [13, 23–25]. However, there is evidence that presenting risk both graphically and numerically can lead to more accurate risk perceptions, to favourable changes in risk factors and can help reduce negative emotions [13]. Evidence for the effects of the ‘heart age’ format is scarce [15]. One recent randomised controlled trial found ‘heart age’ to be more effective than other types of individual’s modelled CVD risk in reducing risk scores [24]. Two studies, one experimental [26] and one qualitative [27] have found decreased perceived credibility for risk scores presented in this way, although the latter found it could still motivate lifestyle change. With increasingly available genetic data it has also been suggested that CVD risk information might be more effective when based on genetic information [28–30]. However two recent systematic review of provision of genetic risk found no clear or consistent evidence that genetic risk communication alone either raises motivation or translates into actual lifestyle change [29, 31]. And many questions remain about the feasibility, accuracy, interpretation and potential harm of providing genetic risk scores [32, 33].

Most qualitative studies exploring the use of CVD risk scores have focused on general practitioners and the consultation process [34–37]. The small number of studies involving the general public have focused on their immediate reaction to risk information in order to identify either their preferences regarding different formats of presentations [38], or their prediction of its potential impact on their future decision making [39]. Only one recent qualitative study focused on the impact of providing genetic risk information [40].

The Information and Risk Modification Trial (INFORM) [41] is a randomised controlled trial comparing the impact of providing phenotypic and genetic coronary heart disease (CHD) risk scores, alongside web-based lifestyle advice. In addition to objective and self-report measures at baseline and follow up, the study also included face-to-face interviews and focus groups which took place throughout the trial. We aimed to explore participant responses to different types of risk information as well as considerations involved in intentions to change or not to change lifestyle and attempts to implement these intentions. This paper reports the results from this qualitative study nested within that trial.

## Methods/Design

### The trial

The design and methods of the INFORM study are described in detail elsewhere [41]. Briefly, INFORM is a parallel-group, open randomised trial in which male and female blood donors with no previous history of CVD aged 40–84 years from across England, who took part in the INTERVAL study [42], were allocated to either no intervention (control group, group 1), or to one of three active intervention groups: lifestyle advice only (group 2); lifestyle advice plus information on estimated 10-year CHD risk based on phenotypic characteristics (group 3); and lifestyle advice plus information on estimated 10-year CHD risk based on phenotypic and genetic characteristics (group 4). Lifestyle advice consisted of three sessions of interactive, tailored web-based information [41].

### The intervention

Participants in all three intervention groups were provided by email with a link to a web-based lifestyle intervention for CHD prevention based on an intervention that was originally developed for the Heart to Health study [43]. The lifestyle intervention consisted of three sessions of interactive, tailored information (up to three hours of interventional contact) with goal setting at the end of each session and some interactive information to help participants overcome individual challenges preventing them from changing their lifestyle.

Following a ‘welcome page’, participants from groups 3 and 4 received information about their risk in a format described below, while participants from group 2 (lifestyle only), after receiving some general information about CHD risk and the potential of lifestyle changes to reduce it, went directly to the lifestyle advice modules (either diet, physical activity or smoking) in which they could choose which modules they wanted to do.

All the participants in the two intervention groups that received risk scores, received a link to a website where their risk scores were presented in the following four ways (participants in group 4 received these four types of presentation twice, once for the phenotypic score and once for the genetic one):

1. Absolute risk of having either a fatal or nonfatal CHD event during the next 10 years, as a percentage with accompanying text explaining what this means (“Your risk of having coronary heart disease in the next 10 years is 31%. This means that approximately 31 out of 100 women like you (31%) will experience coronary heart disease in the next 10 years”).
2. A visual representation of the risk. For that purpose we used a thermometer with three colours, red for high risk above 20%, yellow for moderate risk above 10%, and green for risk under ten percent. An arrow indicated the level of risk. When designing the thermometer we took into account the recommendations by NICE guidelines [44] that advise offering statins to people with a 10 year risk >10% and the AHA guidelines advise offering statins to people with a 10 year risk >7.5%. The low risk (indicated as green) was defined as below 7.5% and then graded to orange (between 7.5–10%) and then graded to red at 20% and above.
3. Peer comparative risk which is the risk of someone who is the same age and sex and has healthy lifestyle-related factors known to be associated with CHD risk.
4. To help people to understand what their absolute risk means we expressed their absolute risk in terms of ‘heart age’. For the purposes of this study, an individual’s ‘heart age’ is defined as the age in which someone with healthy risk factors will have (according to existing population reference values) the same CVD risk which this individual has now [41].

We have described the calculation of the phenotypic and genetic risk scores in detail elsewhere [41]. As we state there: ‘we have included the following set of variables: age, total cholesterol (mmol/l), HDL cholesterol (mmol/l), antihypertensive medication (self-reported yes/no), current smoking (self-reported yes/no) and diabetes mellitus (self-reported yes/no. We chose CHD instead of CVD as the outcome for the risk scores since the majority of genomic loci known to relate to CVD are associated with CHD (rather than stroke) [41]. The Heart Age for phenotypic risk score was derived from the same general formula as used for the absolute risk score, rearranged such that age is unknown, other risk factors are normal and absolute risk is that of the participant in question. The definition of “normal” was based on the following profile: not a current smoker, does not have diabetes, not using antihypertensive medication, total serum cholesterol = 4.6548 mmol/l and HDL cholesterol = 1.1637 mmol/l [23]. Healthy lifestyle-related factors were defined as follows: a) current non-smokers (i.e. never smoked and former smokers) [33]; b) moderate levels of alcohol consumption (one or more units a week but not more than fourteen units a week; 1 unit = 8 g) [33]; c) consumption of fruit and vegetables (more than 400 g) [34]; d) consumption of fish (a portion size of 140 g cooked weigh (20 g/day) [35]; e) consumption of red meat (≤6 portions a week, equivalent to ≤500 g of cooked weight or 71 g per day [36]; f) physical activity (not inactive, at least half an hour of leisure-time activity a day) [33]; g) body mass index (BMI) <25 kg/m2’.

In line with the phenotypic risk score, we estimated mathematical CHD functions to predict 10-year risk of CHD using a similar general formula. For the absolute genetic risk score, this formula was plugged with the same baseline cumulative hazard as the absolute phenotypic risk score, individual lnGRS and the mean lnGRS. For the purpose of ‘heart age’, normal lnGRS was defined as the sex-specific 10th centile (<1.043 for males, <1.045 for females). This calculation meant that participants could have received heart age that was older or younger than their actual age [41].

All forms of presentation were based on evidence regarding the effectiveness of methods for communicating CHD risk estimates which we described above and after consulting with layperson PPI (Patients and Public Involvement in Research) representatives. Examples of a page with all formats of presentations appear in Additional file 1A (for phenotypic risk score) and Additional file 1B (for genetic risk scores). A time frame of the next 10 years was selected in line with timeframe used in NHS Health Checks and the Framingham risk score that we used for calculation of phenotypic risk score [45]. A use of shorter time frame than 10 years (e.g. 5 years) usually provides a small absolute risk estimates especially among those who are younger.

### The qualitative data collection

We sent invitations to take part in the interviews and focus groups to a purposive sample selected from participants who had given their consent to be approached about the qualitative study when completing the online consent form to take part in the trial. We recruited a diverse sample in terms of sex and age. However, in order to sample participants who could provide the richest information relating to our main research questions, we recruited mainly participants who received risk scores, with over representation of participants with medium to high risk (who in theory may have stronger motivation to change lifestyle). From every 100 participants who were recruited to the trial and expressed willingness to take part in the study we sent invitations to participants with an absolute risk (either phenotypic or genetic or both) of above 10% and to participants whose ‘heart age’ (phenotypic or genetic) was at least two years older than their real age. In the earlier stages of recruitment (the first 200 participants) we additionally recruited several participants with an absolute risk between 5–10%. Our reason for choosing this sampling strategy was that the risk score of people who have high absolute risk score and/or a ‘heart age’ older than their chronological age may have an additional incentive/motivation for change because it carries a warning message. Given the geographic spread of the trial participants all over England, we identified two large cities in the North of England that had enough participants to form a focus group that can be located within a realistic driving distance for each participant. The rest were of the participants interviewed individually. Participants took part in either the personal interviews or focus groups, but not both. GS, an experienced qualitative researcher, conducted all the interviews following a topic guide. Each interview lasted between 30–45 min and covered issues related to the participants’ understanding of CVD risk, their reaction to the risk score, their intentions to change behaviour, their attempts at actually changing behaviour if present, and other aspects of their experience of taking part in the trial. GS and BS conducted the focus groups. They lasted between 60–90 min and mainly covered issues related to the formats of presentation of risk scores, and communication and understanding of CVD risk. Accordingly, in the focus groups, after discussing their personal experience of the trial, we provided the participants with a copy of fictional risk scores page, in the same format used in the website in order to discuss preferences and understandings of specific formats of presentations.

### Analysis

We undertook thematic analysis of the qualitative data coding transcripts using NVivo software. To increase the rigour and validity of the analysis, and as a form of triangulation, three members of the team (GS, BS and JUS) developed the coding tree. A subset of 5 transcripts was then coded separately by each of these three researchers and disagreements discussed between them in order to reach consensus about interpretation and indexing. The same thematic framework was applied to the interviews and focus groups. The final coding tree can be seen as Additional file 2. The coding (indexing of excerpts of the data into different categories or codes) for the remainder of the transcripts was conducted by one researcher (GS) following the principles discussed and agreed between the three researchers. This was followed by an interpretation stage of reading through the coded categories and defining the main concepts and mapping the ways in which different parts of the data were related to each other. We sent a summary of the findings (a short summary of each of the three main themes described below) to all qualitative participants by email as a form of further validation and their comments were taken into account when writing the final draft of the manuscript.

## Results

### Interviews and focus groups recruitment

Forty two interviews were conducted between June 1^st^ and October 1^st^ 2015. Most participants were from London, East Anglia and the East Midland areas although a small number were from North West England. Interviews were conducted between 1 and 134 days (median: 40 days) after participants had first accessed the website (based on information collected by the website). Two focus groups, one with six participants and one with seven participants we conducted. The median time between participants first accessing the website and taking part in the focus group was 101 days. One of the participants asked at a later date to be withdrawn from the study and for the related data to be withdrawn as well and we did so regarding this qualitative interview as well.

The characteristics of participants are shown in Table 1 (other baseline information of the participants in the qualitative study appear in Additional file 3) .

**Table 1:** Characteristics of participants

	Interviews	Focus group
	*n* = 41	*n* = 13
Gender		
Male	23	9
Female	18	4
Age (years)		
40–49	8	3
50–59	15	4
60–69	13	5
70–80	5	1
Study group		
4 (genetic + phenotypic risk + lifestyle advice)	22	8
3 (phenotypic risk + lifestyle advice)	15	5
2 (lifestyle only)	4	0
Mean (range) phenotypic risk score (% 10 year risk)		
Male	12.6 (4–62)	6.6 (4–12)
Female	4.5 (1–11)	0.9 (1–2)
Marital status		
Married	28	10
Separated or Divorced	3	1
Widowed	3	0
Single	7	2
Level of Education		
No Formal education	1	0
Secondary education	17	5
University education	23	8

### Findings

Three main themes were identified. First, there are various challenges which limit the potential of risk information to generate concern in people about their existing lifestyle. The second theme identifies the advantages of the ‘heart age’ format in generating concern about sub-optimal lifestyle and awareness of the need to improve it. The third theme is the impact of the website-based lifestyle intervention on intentions and attempts to make moderate lifestyle changes regardless of concerns about the CHD risk score.

1. The challenges limiting the potential of risk information to generate concern about CHD risk and existing lifestyle
  Most of the participants were not concerned about their risk scores but rather were happy with them, or at least felt fine about them. This finding may be expected for participants who received a risk score that was optimal for their age. However, about two thirds of the interviewees who received risk scores had a ‘heart age’ that was older than their chronological age, (i.e. their risk score was higher than someone of the same age and sex with optimal risk factors associated with CHD risk). Despite this, only a minority of these participants were concerned about their risk. Several explanations for this lack of concern about the risk score emerged:
  ➢ Partial noticing and understanding of the risk scores: four interview and two focus group participants did not remember receiving risk scores at all (our website records indicated that all the interview and focus group participants from groups 3 and 4 had accessed their risk score page). Four others had only a vague recollection of the risk score. Additionally, most of those who did remember a risk score were able to recall only one format of presentation, typically an absolute risk score expressed as a percentage. Noticing a percentage risk score without noticing any additional comparative information, meant that whether this was perceived as a high or low risk was largely dependent on the participants’ own preconceptions or on other lay persons opinion. For example, a woman in her early 70s with a phenotypic risk score of 9% remembered she received a risk score of 8%. When asked how did she feel about it she said: “I was quite concerned but my husband didn’t think was very high!” and later added that she was convinced by him that it was not high. Another participant, also female with a phenotypic risk score of 11 thought it was a ‘decent score’. She did not notice her ‘heart age’ which was eight years older than her real age.
  ➢ Overestimation of own risk before the intervention: Many of the participants held overestimating preconceptions about their risk. For example, while the mean of the actual phenotypic risk score given to male interviewees was 11.2%, their mean self-predicted score at baseline was 33.1%. Among female participants this was even more evident, with a mean calculated risk score of 3.5% and a mean self-predicted score of 29.5%.
  ➢ High threshold for what is a high risk: when we asked participants who thought that their risk score was fine what would constitute a high risk in their view, 11 referred to 50% (another commonly mentioned threshold was 10% although there were participants who could not name any threshold). Given this, it is not surprising that risk scores of 10% or lower, and for some over 20%, were not considered concerning. The question of what is a high risk is of course subjective, but some of the explanations given by some participants as to why they felt a risk below 50% was not concerning were quite telling and suggested an vague understanding of probability and risk. For example, one participant commented: *‘[If the risk score is] over half way I should probably do something about it*’ (male participant in his 60s, with phenotypic risk score of 12% and genetic risk score of 13%), another said that ‘*to be in the top half sounds bad’* (female interviewee in her 40s with a phenotypic and genetic risk score of 1%). One participant said specifically that ‘*I tend to interpret it as being obviously a percentage of the population, so 5% risk to me is low. If it was like 50% then that’d be different*’ (male interviewee in his 50s with a phenotypic risk score of 8 and genetic risk score of 6).
  In addition to the general limitations of risk scores, genetic risk scores seemed to be particularly irrelevant to participants. Two thirds of the interviewees who received genetic and phenotypic risk score either didn’t remember them at all or remembered only one risk score and did not remember whether or not it was genetic. The participants also did not understand the meaning of genetic risk score, and those who did notice it interpreted its meaning in a fatalistic way. A participant in one of the focus groups for example commented:
  “I’m just thinking if you have a high genetic risk it’s in your genes, yes lifestyle has an impact but you’ve got it in your genes, what happens then if you die because of the genes? You’ve in a sense exercised yourself potty, deprived yourself of all your nice treats but you’ve still had the same end result. You might as well have enjoyed it and gone!”
  Another participant in a focus group commented that if the genetic risk is high maybe the only option is to go for ‘gene therapy’. A small number of participants had a high genetic risk score (reflected in a heart age older than their chronological age) and a low phenotypic risk score. They found the information particularly unhelpful as they felt that they had a close to optimal lifestyle and their phenotypic risk score and phenotypic heart age confirmed it, and felt they could not do anything about the high genetic risk score. One of them, the most extreme example we had in our sample in term of this disparity between the two risk scores, was a woman in her mid 50s with a phenotypic heart age of 49% and a genetic heart age of 63. She said she was confused, commenting: “it didn’t explain why it would be that way so I mean, I haven’t sort of lost sleep over it but I did kind of think why basically, why should it be that way?”
2. The powerful message carried by an older ‘heart age’
  Notwithstanding the limitations of risks scores in general, ‘heart age’ stood out as a type of risk score presentation that, if noticed, and if higher than the chronological age, communicated a powerful message about how far from optimal the participant’s lifestyle was. About a quarter of the interviewees were concerned about their risk score and almost all of them had noticed their ‘heart age’ and were concerned primarily about their ‘heart age’. This is despite the fact that some of them had a relatively high (above 20) percentage risk score.
  For example, one participant, a male in his 50s, who received a phenotypic risk score of 23% and a ‘heart age’ that was 24 years older than his chronological age, commented that ‘*it was the heart age that jumped out at me […] it was the heart age that really shook me’*.
  For some, the ‘heart age’ also carried with it additional symbolic messages. For example, one participant in his late 50s was particularly concerned that his ‘heart age’ was that of retirement age (65), while he was still in full time work: ‘*It is a spur to be told that your risk is that of somebody who’s retired so it just wakes me up to the fact that I’ve got to be more active*’.
  Concerns about high ‘heart age’ did not automatically create an intention to change lifestyle (and, in one case, created an initial fatalistic reaction), but for some participants it was a serious ‘wake up call’ that encouraged them to make changes to their lifestyle. One participant in particular reported making quite a radical change to his lifestyle after finding out that his ‘heart age’ was 15 years older than his chronological age. He joined a commercial weight-loss scheme, lost about 9 kg by the time of the interview, added wholegrain products to his diet, started walking for half an hour or more at least three times a week and cut out sweets and desserts.
  Other participants who were concerned about their ‘heart age’ attempted to make more moderate lifestyle changes such as having less white bread or increasing their fruit and vegetable intake. For example, a male interviewee, in his late 50s, with a phenotypic risk score of 11% and a heart age that was 6 years older than his own age, explained the reasons for the lifestyle changes he made following the study (stopped drinking sugary drinks) in the following way, referring specifically to the ‘heart age’ format or presentation: “the study sort of nails things that you know about these things, and it sort of nails it down, hang on a minute, you’re actually running out of time here and you’ve got to do it”.
3. The impact of the website-based lifestyle intervention on intentions and attempts to make moderate lifestyle changes
  Reports of attempts to change lifestyle were, however, not limited to participants who were concerned about their high heart age. In fact, more than two thirds of the interviewees, including more than half of the interviewees who had risk scores that were low or moderate for their age reported not only intending and attempting to implement some lifestyle changes during the study, but actually being able to maintain the change at the time of the interview or focus group (as for all the findings, these are the results from the qualitative element of the study). Typically, these changes were modest. Among the lifestyle changes mentioned by participants were the following: having salad for lunch; adding one session of walking in the weekends to pre-existing walking sessions during weekdays; cutting out chocolate and wine; having one more fish meal a week; buying a pedometer and trying to increase the daily step count; having some alcohol free evenings.
  Some participants made more radical changes regardless of their risk score. One interviewee who was in the group receiving lifestyle advice only, a woman in her 50s, reported making as radical a change to her lifestyle as the person concerned by his ‘heart age’ mentioned above. She stated that she had lost about 8 kg, after some drastic changes to her diet, moving to wholemeal food, and increasing her number of daily steps from 7000 to 12,000.
  When we asked participants what stimulated them to make these changes in their lifestyle, many explained that they were already aware of the need to do something and had some idea of what they could do to change their lifestyle but the study acted as a ‘reminder’; ‘a trigger’; ‘a nudge’; a tool to ‘refocus awareness’. One of the participants, a male in his late 60s with a phenotypic risk score of 9% commented about his decision to consume more fruits and vegetables and cut on his alcohol consumption in the following words: “I think goal setting helped me make the changes because I was aware that both [fruit and veg consumption and alcohol consumption, G.S] were wrong anyway, or were out of balance, so it was quite an incitement to do that, to change”
  For others, the change in lifestyle was a combination of the study being a trigger encouraging them to do something they already intended to do and a wish to help the study: ‘*it gave me the kick that I needed, and also it gives you that kind of, ‘Oh, I’m helping. I’m involved in a study*.’ (a female interviewee in her 40s with a phenotypic risk score of 1% who did not receive genetic risk score).
  The lifestyle advice website was viewed as instrumental in facilitating the change for participants. While most participants were familiar with the majority of the information and advice, some participants reported learning new information about some aspects of healthy lifestyle, particularly regarding diet. Many participants found the interactive format of the website, the personal advice, the goal setting and more generally the autonomy that they felt they had when deciding what changes to do and when and how to do them quite appealing. They explained that this approach allowed them to attempt making changes in their own pace and terms.
  A particular repeated comment referred to the non-preaching nature of the lifestyle website, in comparison to interventions that participants had encountered in the past. Their memories of other interventions were that they were ‘lectured’ by them, thus making them defensive and reluctant to engage. One interviewee, a woman in her 60s with a phenotypic risk score of 6% and a ‘heart age’ which was lower than her real age, commented, for example:
  “It gives you an opportunity to set your own target. So you can make it as big a step or as small a step as you like. It’s flexible and there’s nobody going to be there with a, you know, a knife in your back if you don’t do it, you know, there’s nobody looking over your shoulder and saying, ‘You didn’t do such and such a thing’.
  Another interviewee, a man in his 50s, also with a phenotypic risk score of 6% and with a ‘heart age’ which was lower than his real age, commented in a similar fashion:
  I thought it was pretty good, it was better than I expected. I did wonder if it was going to lecture me or try to frighten me, but I thought it was quite easy to use, it was clear, the information was there and it didn’t sort of judge you or anything. So I thought it was quite good and I did the whole thing and that was fine, yeah.
  Not all participants attempted to change their lifestyle. There was a small group of participants who felt that they did not learn anything new from the advice given in the website and that their lifestyle was already close to optimal. However, none of the participants reported that they intended to do less than they were doing before the study because of receiving a risk score that was lower than they expected. In other words, we did not find evidence for false reassurance.

## Discussion

### Principal findings

The findings of this study reflect some of the challenges and opportunities for using risk scores and lifestyle advice as part of a strategy to reduce the burden of cardiovascular disease. In relation to risk scores, the study identifies some of the barriers limiting the potential of risk scores to create concern about an individual’s probability of having CHD. Risk scores were frequently overlooked and not meaningful, particularly genetic risk scores, and significantly lower than prior perceptions.

Despite these limitations, a risk score in the format of ‘heart age’, if noticed and if higher than the chronological age, can communicate a powerful message that an individual’s CHD risk is not optimal. Regardless of risk-scores, a user friendly, interactive, goal setting based lifestyle advice website appeared to motivate individuals to attempt to make modest changes in lifestyle [46].

### Results in context

Many of the challenges of understanding and interpreting risk scores have been reported previously [47, 48]. Our study shows, however, that using different risk score presentation formats together, does not necessarily eliminate these challenges. Providing multiple types of presentation does not automatically translate into increased understanding. Individuals struggle to absorb the many different types of information, and many of them notice only one type of presentation and rarely more than two. Combined with a preconception about what constitutes a high risk, noticing only one type of risk information, is not necessarily followed by a heightened concern about risk.

However, if individuals notice their ‘heart age’ then even without any additional information it can provide a ‘full message’ about non optimal lifestyle. Age is a more familiar, concrete and absolute concept than probability and since people know their chronological age, it is quite simple to communicate to them a message about their lifestyle being less than optimal. Another main advantage of a ‘heart age’ is that it can communicate a message about non optimal lifestyle even at younger age, when the absolute risk is likely to be quite low, thus potentially encouraging younger people to change their lifestyle at an age when lifestyle changes, if sustained, can have a greater impact on decreasing risk of CHD. These results are consistent with a recent randomised controlled trial [24] that found ‘heart age’ to be more effective than other types of individual’s modelled CVD risk in reducing risk scores. They are also consistent to a large extent with a previous qualitative study that found that heart age can motivate lifestyle change [27]. We did not find decreased perceived credibility to this format of presentation of risk scores reported by that and other study [26], although if much higher than the actual age, ‘heart age’, like the genetic risk scores, can lead to some fatalistic views about CVD risk.

There is some evidence that fear can have a positive impact on attitudes and behaviour [49]. Some psychological theories of health-related behaviour change postulated that perceived risk can play a central role in adoption of health-related behaviours [50]. For example, in the Health Belief Model (HBM), this is reflected in the concept of perceived threat, a combination of perceived susceptibility and perceived severity [51]. This study however indicates that other factors might be at least as relevant for adoption of lifestyle changes which may be in line with more recent health behaviour change theories such as intention-implementation. This finding is supported by the fact that many participants attempted moderate changes to their lifestyle while being indifferent (or happy) about their risk.

### Implications for clinical practice and future research

Risk information is being provided with increasing frequency in routine consultations and as part of screening and risk reduction programmes such as NHS Health Checks [52]. Given the difficulties people appear to have with understanding risk scores when presented in multiple formats, a simplified format of risk score presentation may be clearer, especially if used in the format of ‘heart age’. Other forms of risk information, such as the percentage risk of developing disease over the next 10 years used widely within primary care and within the NHS health checks, may be less useful and limited by people’s tendency to overestimate their risk and intrinsic difficulties understanding numbers and probabilities. Genetic risk scores also appear to be less meaningful and can lead to fatalistic responses as was very recently suggested [29]. ‘Heart age’, however, can also lead to a fatalistic response as real age is irreversible and some participants interpreted the heart age to imply that they are older than their age and that ‘time is running out’. Any strategy using either ‘heart age’ or genetic risk will, therefore, need to put emphasis on clarifying that the overall risk can still be reduced following a change in lifestyle. For that purpose, a detailed explanation about the increased risk as a result of individual’s genetic predisposition may be simpler to understand than calculating genetic risk scores.

The limited understanding and impact of the online risk score information in this study suggests that some individuals may benefit from risk information being provided face-to-face as part of a consultation with a healthcare practitioner. In such a consultation, the concept of risk can be further discussed and interpreted and the practitioner can answer patients’ questions and clarify misunderstandings. However, previous studies have shown that participant recall of risk is still very low even after being given risk information face-to-face [16]. The findings from this study also suggest that when conducting such consultations it is important to be aware of the preference of some individuals to have more control on the pace and nature of the lifestyle change without feeling pushed too much by threatening messages and overemphasising of the risk. Others may find an interactive, goal setting based internet site a useful trigger to start the process of lifestyle change. This may of course also be a cheaper alternative to face-to-face consultations [46].

### Limitation and strengths

In addition to general limitations on the representativeness of small purposive samples in qualitative studies, the participants in this trial may be less representative of the general population. Our participants are blood donors and participants in the INTERVAL study who are likely to have better knowledge of and higher commitment to healthier lifestyles. For example, the mean phenotypic risk score of the participants of this study was 12.6% for men and 4.5% for women while the mean phenotypic risk score for participants in the population-based EPIC-Norfolk study (from which risk score equations were derived) was 15.2% for men and 6.7% for women respectively. The participants were also quite highly educated with over half of the sample having had a university education. These sample demographics should be taken into account when interpreting our findings; however they may actually add extra strength to some of them. It is arguable, for example, that the limitations in understanding and interpreting the risk scores are likely to be even more considerable in people with lower levels of education.

The relative short time scale of the study additionally meant that we were not able to explore in depth the challenges involved in maintaining lifestyle changes over the longer term. We therefore cannot tell how long any changes in lifestyle reported by participants may last and what their impact might be on CHD risk. Some of these questions may be addressed by the quantitative results of the INFORM trial but others may require longer studies.

## Conclusion

Provision of CHD risk information, for example as part of NHS Health Checks in the UK is unlikely to have a significant impact in and of itself, especially if absolute risk is presented as a percentage. Genetic risk information alone is also unlikely to motivate engagement in risk-reducing behaviours. Using risk scores in the format of ‘heart age’ can overcome limitations of other formats of CHD risk score presentation and interactive personalised lifestyle advice can generate motivation for lifestyle advice even when participants are indifferent about their risk scores.

## Additional files

Additional file 1:
**A**. An example of presentation of phenotypic coronary heart disease risk score. **B**. An example of presentation of genetic coronary heart disease risk score. (ZIP 59 kb)
Additional file 2: Basic Coding Tree INFORM Qualitative Study (interviews and focus groups). (DOCX 15 kb)
Additional file 3: Baseline information of the participant in the qualitative study. (DOCX 14 kb)

## References

[1] World Health Organization. Global status report on noncommunicable diseases 2010. 2011. http://whqlibdoc.who.int/publications/2011/9789240686458_eng.pdf?ua=1. Accessed 3 Nov 2015.

[2] Mendis S, Puska P, Norrving B. Global atlas on cardiovascular disease prevention and control. World Health Organization; 2011. http://whqlibdoc.who.int/publications/2011/9789241564373_eng.pdf. Accessed 3 Nov 2015.

[3] GBD 2013 Mortality and Causes of Death Collaborators (2015). Global, regional, and national age-sex specific all-cause and cause-specific mortality for 240 causes of death, 1990-2013: a systematic analysis for the Global Burden of Disease Study 2013. Lancet.

[4] Mathers CD, Loncar D (2006). Projections of global mortality and burden of disease from 2002 to 2030. PLoS Med.

[5] Satish M (2005). Coronary heart disease in clinical practice.

[6] Gaziano M, Ridker PM, Libby P, Bonow RO, Mann DL, Zipes DP, Libby P (2012). Primary and secondary prevention of coronary heart disease. Braunwald’s heart disease. A textbook of cardiovascular medicine.

[7] Mozaffarian D, Capewell S (2011). United Nations’ dietary policies to prevent cardiovascular disease. BMJ.

[8] Anderson KM, Odell PM, Wilson PW, Kannel WB (1991). Cardiovascular disease risk profiles. Am Heart J.

[9] Hippisley-Cox J, Coupland C, Vinogradova Y, Robson J, May M, Brindle P (2007). Derivation and validation of QRISK, a new cardiovascular disease risk score for the United Kingdom: prospective open cohort study. BMJ.

[10] Woodward M, Brindel P, Tunstall-Pedoe H (2007). Adding social deprivation and family history to cardiovascular risk assessment: the ASSIGN score from the Scottish Heart Health Extended Cohort (SHHEC). Heart.

[11] Conroy RM, Pyorala K, Fitzgerald AP, Sans S, Menotti A, De BG (2003). Estimation of ten–year risk of fatal cardiovascular disease in Europe: the SCORE project. Eur Heart J.

[12] Ridker PM, Buring JE, Rifai N, Cook NR (2007). Development and validation of improved algorithms for the assessment of global cardiovascular risk in women: the Reynolds Risk Score. JAMA.

[13] Waldron CA, van der Weijden T, Ludt S, Gallacher J, Elwyn G (2011). What are effective strategies to communicate cardiovascular risk information to patients A systematic review. Patient Educ Couns.

[14] Cooney MT, Vartiainen E, Laatikainen T, De Bacquer D, McGorrian C, Dudina A, Graham I (2012). Cardiovascular risk age: concepts and practicalities. Heart.

[15] Groenewegen KA, den Ruijter HM, Pasterkamp G (2016). Vascular age to determine cardiovascular disease risk: a systematic review of its concepts, definitions, and clinical applications. Eur J Prev Cardiol.

[16] Usher-Smith JA, Silarova B, Schuit E (2015). Impact of provision of cardiovascular disease risk estimates to healthcare professionals and patients: a systematic review. BMJ Open.

[17] Sheridan SL, Viera AJ, Krantz MJ (2010). The effect of giving global coronary risk information to adults: a systematic review. Arch Intern Med.

[18] Janz NK, Becker MH (1984). The health belief model: a decade later. Health Educ Q.

[19] Rogers RW (1975). A protection motivation theory of fear appeals and attitude change. J Psychol.

[20] Ajzen I (1991). The theory of planned behavior. Organ Behav Hum Decis Process.

[21] Schwarzer R (2008). Modeling health behavior change: How to predict and modify the adoption and maintenance of health behaviors. Applied Psychology.

[22] Gollwitzer PM (1999). Implementation intentions: strong effects of simple plans. American Psychologist.

[23] Soureti A, Hurling R, Murray P, van Mechelen W, Cobain M (2010). Evaluation of a cardiovascular disease risk assessment tool for the promotion of healthier lifestyles. Eur J Cardiovasc Prev Rehabil.

[24] Lopez-Gonzalez AA, Aguilo A, Frontera M, Bennasar-Veny M, Campos I, Vicente-Herrero T, Tomas-Salva M, De Pedro-Gomez J, Tauler P. Effectiveness of the Heart Age tool for improving modifiable cardiovascular risk factors in a Southern European population: a randomized trial. Eur J Prev Cardiol. 2015;22(3):389-96. 10.1177/2047487313518479 24491403

[25] Zipkin DA, Umscheid CA, Keating NL (2014). Evidence-based risk communication: a systematic review. Ann Intern Med.

[26] Bonner C, Jansen J, Newell BR, Irwig L, Teixeira-Pinto A, Glasziou P (2015). Is the ‘heart age’ concept helpful or harmful compared to absolute cardiovascular disease risk? An experimental study. Med Decision Making.

[27] Bonner C, Jansen J, Newell BR, Irwig L, Glasziou P, Doust J, et al. I Don’t Believe It, But I’d Better Do Something About It: Patient Experiences of Online Heart Age Risk Calculators. J Med Internet Res. 2014;16(5):e120. 10.2196/jmir.3190 PMC4026572 24797339

[28] Thanassoulis G, Vasan RS (2010). Genetic cardiovascular risk prediction will we get there?. Circulation.

[29] Hollands GJ, French DP, Griffin SJ, Prevost AT, Sutton S, King S, Marteau TM (2016). The impact of communicating genetic risks of disease on risk-reducing health behaviour: systematic review with meta-analysis. BMJ.

[30] Kullo IJ, Jouni H, Austin EE (2016). Incorporating a genetic risk score into coronary heart disease risk estimates: effect on low-density lipoprotein cholesterol levels (the MI-GENES clinical trial). Circulation.

[31] Li SX, Ye Z, Whelan K, Truby H (2016). The effect of communicating the genetic risk of cardiometabolic disorders on motivation and actual engagement in preventative lifestyle modification and clinical outcome: a systematic review and meta-analysis of randomised controlled trials. Br J Nutr.

[32] Knowles (2012). Randomized trial of personal genomics for preventive cardiology. Design and challenges. Circ Cardiovasc Genet.

[33] Janssens C (2014). The hidden harm behind the return of results from personal genome services: a need for rigorous and responsible evaluation. Genet Med.

[34] Wan Q, Harris MF, Zwar N, Vagholkar S, Campbell T (2010). Prerequisites for implementing cardiovascular absolute risk assessment in general practice: a qualitative study of Australian general practitioners’ and patients’ views. J Eval Clin Pract.

[35] Wan Q, Harris M, Zwar N, Vagholkar S, Kemp L, Campbell T (2010). Experience in implementation of cardiovascular absolute risk assessment and management in Australian general practice. International journal of clinical practice. Int J Clin Pract.

[36] Liew SM, Blacklock C, Hislop J, Glasziou P, Mant D (2013). Cardiovascular risk scores: qualitative study of how primary care practitioners understand and use them. Br J Gen Pract.

[37] Bonner C, Jansen J, Mckinn S, Irwig L, Doust J, Glasziou P, Hayen A, McCaffery K (2013). General practitioners’ use of different cardiovascular risk assessment strategies: a qualitative study. Med J Aust.

[38] Hill S (2010). Absolute risk representation in cardiovascular disease prevention: comprehension and preferences of health care consumers and general practitioners involved in a focus group study. BMC Public Health.

[39] Sheridan SL, Behrend L, Vu MB, Meier A, Griffith JM, Pignone MP (2009). Individuals’ responses to global CHD risk: a focus group study. Patient Educ Couns.

[40] Middlemass JB, Yazdani MF, Kai J, Standen PJ, Qureshi N (2014). Introducing genetic testing for cardiovascular disease in primary care: a qualitative study. Br J Gen Pract.

[41] Silarova B, Lucas J, Butterworth AS, Di Angelantonio E, Girling C, Lawrence K, … & Griffin S. Information and Risk Modification Trial (INFORM): design of a randomised controlled trial of communicating different types of information about coronary heart disease risk, alongside lifestyle advice, to achieve change in health-related behaviour. BMC Public Health. 2015;15(1):1. 10.1186/s12889-015-2192-5 PMC4562192 26345710

[42] Moore C, Sambrook J, Walker M, Tolkien Z, Kaptoge S, Allen D, Mehenny S, Mant J, Di Angelantonio E, Thompson SG, Ouwehand W (2014). The INTERVAL trial to determine whether intervals between blood donations can be safely and acceptably decreased to optimise blood supply: study protocol for a randomised controlled trial. Trials.

[43] Sheridan SL, Draeger LB, Pignone MP, Sloane PD, Samuel-Hodge C, Finkelstein EA (2013). Designing and implementing a comparative effectiveness study of two strategies for delivering high quality CHD prevention: methods and participant characteristics for Heart to health study. Contemp Clin Trials.

[44] National Institute for Health and Care Excellence. Lipid modification: cardiovascular risk assessment and the modification of blood lipids for the primary and secondary prevention of cardiovascular disease (CG181). 2014. http://www.nice.org.uk/guidance/cg181/resources/guidance-lipid-modification-cardiovascular-risk-assessment-and-the-modification-of-blood-lipids-for-the-primary-and-secondary-prevention-of-cardiovascular-disease-pdf. Accessed 2 Nov 2016. 25340243

[45] D’Agostino RB, Vasan RS, Pencina MJ, Wolf PA, Cobain M, Massaro JM (2008). General cardiovascular risk profile for use in primary care: the Framingham Heart Study. Circulation.

[46] Keyserling TC, Sheridan SL, Draeger LB, Finkelstein EA, Gizlice Z, Kruger E, Johnston LF, Sloane PD, Samuel-Hodge C, Evenson KR, Gross MD, Donahue KE, Pignone MP, Vu MB, Steinbacher EA, Weiner BJ, Bangdiwala SI, Ammerman AS (2014). A comparison of live counseling with a web-based lifestyle and medication intervention to reduce coronary heart disease risk: a randomized clinical trial. JAMA Intern Med.

[47] Lipkus IM (2007). Numeric, verbal, and visual formats of conveying health risks: suggested best practices and future recommendations. Med Decis Making.

[48] Spiegelhalter D, Pearson M, Short I (2011). Visualizing uncertainty about the future. Science.

[49] Tannenbaum MB, Hepler J, Zimmerman RS, Saul L, Jacobs S, Wilson K, Albarracín D (2015). Appealing to fear: a meta-analysis of fear appeal effectiveness and theories. Psychol Bull.

[50] Noar SM, Zimmerman RS (2005). Health Behavior Theory and cumulative knowledge regarding health behaviors: are we moving in the right direction?. Health Educ Res.

[51] Glanz K, Rimer BK, Viswanath K, editors. Health behavior and health education: theory, research, and practice. San Fransisco: Wiley; 2008.

[52] Krogsbøll LT, Jørgensen KJ, Larsen CG, Gøtzsche PC (2012). General health checks in adults for reducing morbidity and mortality from disease: Cochrane systematic review and meta-analysis. BMJ.

