# Supplementary material for: The response to receiving phenotypic and genetic coronary heart disease risk scores and lifestyle advice – a qualitative study
Source: BMC Public Health. 2016 Dec 3;16:1221. doi: 10.1186/s12889-016-3867-2 (PMC5135826; doi:10.1186/s12889-016-3867-2)
Supplement: Additional file 1: — A. An example of presentation of phenotypic coronary heart disease risk score. B. An example of presentation of genetic coronary heart disease risk score. (ZIP 59 kb) [file 12889_2016_3867_MOESM1_ESM.zip › Additional file 1AR1.docx]

**Additional file 1A*.** An example of presentation of phenotypic coronary heart disease risk score.

**Your risk of coronary heart disease based on traditional risk factors**


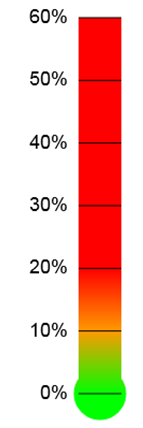


Risk for a woman with a healthy lifestyle

15%

Your risk

31%

**Your risk now**

Your risk of having coronary heart disease in the next 10 years is 31%. This means that approximately 31 out of 100 women like you (31%) will experience coronary heart disease in the next 10 years.

**Your Heart Age**

Your heart age is 83 years. This means that your risk of having coronary heart disease in the next 10 years is about the same as an 83-year-old woman who does not smoke, does not have diabetes and who has normal blood pressure and cholesterol.

**For comparison,** a woman the same age as you who does not smoke, exercises regularly, eats healthily and drinks moderate levels of alcohol has a 15% risk of having coronary heart disease during the next 10 years.

**Remember:**

- A high-risk estimate does not mean that you are destined to have coronary heart disease, and a low-risk estimate does not mean that you are completely without risk of having coronary heart disease.
- The estimate we have given you includes the risk of having a fatal or nonfatal coronary heart disease in the next 10 years – for example, angina (chest pain and discomfort on exercise) or a heart attack.
- For most people, there are numerous ways to reduce the risk of having coronary heart disease.

If you would like to know more about the traditional (coronary heart disease) risk estimates please click on the following link (Frequently asked questions):

**Frequently asked questions**

**Where did these estimates come from?**

For people who have not previously had coronary heart disease, the risk of having coronary heart disease can be calculated using published risk estimates (i.e. formulas) that have been developed using large studies, which followed healthy people for more than 10 years, to see who would have coronary heart disease and who would not. Although these estimates apply well to large populations, they do not apply perfectly to all individuals. We used the following information to calculate your traditional coronary heart disease risk estimate:

1. Your age
2. Your sex
3. Your cholesterol level
4. Your HDL (“good”) cholesterol level
5. The information you provided about whether or not you take medication for high blood pressure
6. Your smoking status
7. The information you provided about whether you have diabetes or not

If any of this information is incorrect, the risk estimate might not be accurate.

**Why might this risk seem different to what a doctor told me in the past?**

You may have been told in the past that your risk of having coronary heart disease was different than the risk reported here. That may be because we were not able to measure your blood pressure during this study (as a doctor would) and, therefore, had to rely only on the information you provided about whether or not you take medication for high blood pressure. Also there are several different scores used for calculation of coronary heart disease risk estimate.

NOTE: The answers to frequently asked questions regarding risk estimates are adapted from a randomised trial of personal genomics for preventive cardiology by Knowles et al. 2012: *Randomized trial of personal genomics for preventive cardiology: design and challenges*; and from a study by Persell et al. 2013: *Electronic health record-based patient identification and individualized mailed outreach for primary cardiovascular disease prevention: a cluster randomized trial*.

*Taken from: Silarova, Barbora, et al. "Information and Risk Modification Trial (INFORM): design of a randomised controlled trial of communicating different types of information about coronary heart disease risk, alongside lifestyle advice, to achieve change in health-related behaviour." *BMC public health* 15.1 (2015): 1.
