# Supplementary material for: The response to receiving phenotypic and genetic coronary heart disease risk scores and lifestyle advice – a qualitative study
Source: BMC Public Health. 2016 Dec 3;16:1221. doi: 10.1186/s12889-016-3867-2 (PMC5135826; doi:10.1186/s12889-016-3867-2)
Supplement: Additional file 2: — Basic Coding Tree INFORM Qualitative Study (interviews and focus groups). (DOCX 15 kb) [file 12889_2016_3867_MOESM2_ESM.docx]

**Additional file 2.** Basic Coding Tree INFORM Qualitative Study (interviews and focus groups)

| **Risk** |
| --- |
| - Previous knowledge or thoughts about risk |
| - - As a result of a previous medical information |
| - - Otherwise |
| - - Did not have any knowledge and did not think about it much |
| - - Family history |
| - - Not a concern |
| - Perception of risk general |
| - Perception of own risk |
| - - Remembering own risk |
| - - Reaction to risk |
| - - - What they think or feel about the risk |
| - Risk lower or what they expected |
| - Risk higher than expected |
| - Other comments about the risk |
| - Discussed it with someone |
| - Question the validity of the risk score |
| - Not concerned |
| - Comments about format of risk presentation |
| - Genetic risk |
| - Comments about the genetic risk score provided to them |
| - Other comments about genetic risk |
| **Lifestyle** |
| - Pre study lifestyle |
| - Diet |
| - PA |
| - Smoking |
| - Alcohol |
| - Previous changes in lifestyle |
| - Diet |
| - PA |
| - Smoking |
| - Alcohol |
| - Changes following the study |
| - Intentions and goals |
| - Diet |
| - PA |
| - Smoking |
| - Other |
| - Do not intend to change anything |
| - Alcohol |
| - Attempts to change lifestyle |
| - Diet |
| - PA |
| - Other (including alcohol and smoking, pedometer) |
| - Barriers |
| - Facilitators |
| - Reasons the study triggered (or not) intentions to change |
| **Website and intervention** |
| - What they learned |
| - Comments about the validity of the information |
| - Comments about the website |
| - Problems, things to improve |
| - Things that work well |
| - Any other comment about the website and intervention |
| **Other** |
| - Comments about previous communication with the healthcare system |
| - Ideas, suggestion, comments and advice about changing lifestyle |
| - Other |
