# Supplementary material for: The response to receiving phenotypic and genetic coronary heart disease risk scores and lifestyle advice – a qualitative study
Source: BMC Public Health. 2016 Dec 3;16:1221. doi: 10.1186/s12889-016-3867-2 (PMC5135826; doi:10.1186/s12889-016-3867-2)
Supplement: Additional file 3: — Baseline information of the participant in the qualitative study. (DOCX 14 kb) [file 12889_2016_3867_MOESM3_ESM.docx]

**Additional file 3 - Baseline information of the participant in the qualitative study**

| **ID** | **Sex** | **Age Group**  **(years)** | **Study Group*** | **Phenotypic**  **Risk (%)** | **Genetic risk (%)** | **Phenotypic heart age** | **Genetic**  **heart age** | **Self- reported Health in general**** | **smoking** | **Self-reported Fruits and veg 5 a day?** | **Self-reported 30 min walking 5 days a week** |
| --- | --- | --- | --- | --- | --- | --- | --- | --- | --- | --- | --- |
| 1 | M | 60-64 | 3 | 6 | N/A | 56 | N/A | Very good | No, never smoked | Yes | Yes |
| 2 | M | 70-74 | 3 | 7 | N/A | 59 | N/A | Very good | No, but smoked before | No | No |
| 3 | M | 55-59 | 3 | 6 | N/A | 55 | N/A | Very good | No, never smoked | No | No |
| 4 | F | 55-59 | 4 | 3 | 2 | 57 | 59 | Very good | No, never smoked | Yes | Yes |
| 5 | M | 70-74 | 4 | 62 | 28 | 112 | 83 | Good | No, but smoked before | Yes | No |
| 6 | F | 60-64 | 3 | 4 | N/A | 61 | N/A | Very good | No, but smoked before | Yes | Yes |
| 7 | M | 55-59 | 3 | 23 | N/A | 81 | N/A | Very good | No, never smoked | Yes | Yes |
| 8 | M | 65-69 | 3 | 9 | N/A | 62 | N/A | Good | No, never smoked | No | Yes |
| 9 | F | 70-74 | 4 | 9 | 13 | 70 | 81 | Very good | No, never smoked | Yes | No |
| 10 | M | 65-69 | 4 | 7 | 18 | 57 | 73 | Very good | No, never smoked | Yes | Yes |
| 11 | M | 65-69 | 3 | 8 | N/A | 60 | N/A | Fair | No, but smoked before | Yes | No |
| 12 | M | 60-64 | 3 | 8 | N/A | 61 | N/A | Very good | No, never smoked | Yes | Yes |
| 13 | F | 65-69 | 3 | 6 | N/A | 65 | N/A | Good | No, never smoked | No | Yes |
| 14 | M | 55-59 | 3 | 14 | N/A | 70 | N/A | Fair | No, never smoked | No | No |
| 15 | M | 60-64 | 4 | 12 | 13 | 67 | 67 | Good | No, never smoked | No | Yes |
| 16 | F | 60-64 | 3 | 11 | N/A | 72 | N/A | Very good | No, but smoked before | Yes | Yes |
| 17 | M | 60-64 | 4 | 25 | 11 | 83 | 65 | Very good | No, but smoked before | No | Yes |
| 18 | F | 75-79 | 4 | 9 | 15 | 70 | 82 | Very good | No, but smoked before | Yes | Yes |
| 19 | M | 45-49 | 4 | 4 | 5 | 49 | 54 | Good | No, but smoked before | No | No |
| 20 | M | 55-59 | 4 | 13 | 8 | 69 | 60 | Good | No, never smoked | No | Yes |
| 21 | M | 55-59 | 3 | 10 | N/A | 64 | N/A | Very good | No, never smoked | No | Yes |
| 22 | M | 75-79 | 4 | 12 | 21 | 67 | 77 | Very good | No, but smoked before | Yes | Yes |
| 23 | M | 55-59 | 4 | 14 | 7 | 70 | 59 | Fair | No, never smoked | No | No |
| 24 | M | 65-69 | 4 | 4.8 | 19.9 | 53 | 73 | Good | No, never smoked | Yes | No |
| 25 | F | 40-44 | 4 | 0.6 | 0.7 | 43 | 49 | Good | No, but smoked before | No | Yes |
| 26 | F | 50-54 | 4 | 1.2 | 3.1 | 49 | 63 | Very good | No, never smoked | Yes | Yes |
| 27 | M | 55-59 | 4 | 8 | 6 | 60 | 57 | Good | No, never smoked | No | No |
| 28 | M | 60-64 | 3 | 10 | N/A | 64 | N/A | Good | No, but smoked before | No | No |
| 29 | M | 55-59 | 3 | 7 | N/A | 59 | N/A | Very good | No, but smoked before | Yes | Yes |
| 30 | F | 40-44 | 3 | 0.9 | N/A | 47 | N/A | Fair | No, never smoked | No | No |
| 31 | M | 65-69 | 4 | 9 | 17 | 63 | 72 | Very good | No, but smoked before | Yes | Yes |
| 32 | M | 55-59 | 4 | 11 | 10 | 65 | 64 | Good | No, never smoked | No | Yes |
| 33 | F | 50-54 | 4 | 3 | 1 | 57 | 52 | Very good | Yes, occasionally | Yes | No |
| 34 | F | 55-59 | 4 | 1.5 | 2.9 | 50 | 62 | Fair | No, but smoked before | No | Yes |
| 35 | F | 55-59 | 4 | 4 | 1 | 61 | 54 | Good | Yes, daily | No | Yes |
| 36 | F | 45-49 | 4 | 0.7 | 0.6 | 45 | 48 | Very good | No, but smoked before | No | Yes |
| 37 | F | 55-59 | 2 | 9.3 | N/A | 70 | N/A | Good | No, never smoked | No | No |
| 38 | F | 50-54 | 2 | 2 | N/A | 51 | N/A | Good | No, but smoked before | Yes | Yes |
| 39 | F | 45-49 | 4 | 0.6 | 0.5 | 43 | 46 | Very good | No, but smoked before | No | Yes |
| 40 | F | 50-54 | 2 | 1 | N/A | 46 | N/A | Very good | No, but smoked before | No | Yes |
| 41 | F | 45-49 | 2 | 1 | N/A | 47 | N/A | Fair | No, never smoked | No | No |
| 42 | M | 50-54 | 3 | 4 | N/A | 50 | N/A | Very good | No, never smoked | No | Yes |
| 43 | M | 55-59 | 4 | 4 | 8 | 49 | 61 | Good | No, never smoked | No | Yes |
| 44 | F | 45-49 | 4 | 0.9 | 0.7 | 46 | 49 | Very good | No, but smoked before | Yes | No |
| 45 | M | 50-54 | 4 | 12 | 7 | 66 | 58 | Very good | No, never smoked | Yes | Yes |
| 46 | M | 60-64 | 4 | 7.2 | 15 | 59 | 70 | Very good | No, never smoked | No | Yes |
| 47 | M | 65-69 | 4 | 6 | 14 | 57 | 70 | Very good | No, never smoked | No | Yes |
| 48 | F | 60-64 | 3 | 2 | N/A | 54 | N/A | Good | No, never smoked | No | Yes |
| 49 | F | 40-44 | 4 | 0.3 | 0.4 | 39 | 44 | Very good | No, never smoked | Yes | Yes |
| 50 | F | 40-44 | 3 | 0.4 | N/A | 39 | N/A | Good | No, but smoked before | Yes | Yes |
| 51 | M | 55-59 | 3 | 7.4 | N/A | 59 | N/A | Good | No, but smoked before | No | Yes |
| 52 | M | 60-64 | 4 | 6 | 13 | 56 | 68 | Good | No, never smoked | Yes | No |
| 53 | M | 70-74 | 3 | 9 | N/A | 62 | N/A | Very good | No, but smoked before | Yes | Yes |
| 54 | M | 60-64 | 4 | 4.5 | 11.4 | 52 | 66 | Very good | No, never smoked | Yes | Yes |
